# P14. Cell-based gene delivery leverages conventional immunotherapy for cancer

**DOI:** 10.1186/2051-1426-2-S2-P5

**Published:** 2014-03-12

**Authors:** C Günther, R Huss

**Affiliations:** 1 apceth GmbH & Co. KG, Ottobrunn, Germany

## 

Current immunotherapies against cancer comprise of target specific antibodies, cellular therapies like activated immune cells (e.g. DCs or NK cells), vaccination strategies, the use of tumour-specific infectious agents like oncolytic viruses or a combination thereof including conventional chemotherapies. All of those approaches attempt to target cancer specific pathways or certain immune response mechanisms, which have to be activated or at least present in the individual patient. This requires the identification of predictive biomarker and / or the execution of large clinical trials. Recent reports have shown that most clinical responses to targeted therapies are still quite unpredictable and yield in large clinical trials to demonstrate any benefit over conventional therapies.

Therefore there is still an unmet medical need to develop new therapies, identify novel target structures in cancer or combine different therapies to optimise the patient's outcome. Cell-based therapies as a pharmaceutical delivery tool for drugs or genes are expected to improve clinical medicine and the inflammatory tumour microenvironment (iTME) could be a novel target. We have combined those to innovative approaches to use mesenchymal stem cells (MSCs) delivering a gene directly into the iTME and activated a cytotoxic prodrug such as Ganciclovir only in the context of the tumour or its metastasis. This increases the local anti-tumour efficacy and reduces unwanted off-target toxicities. A first clinical trial (TREAT-ME1) targeting adenocarcinomas of the GI tract is currently being conducted.

Although the idea to use cells as pharmaceutical compounds is not new, the clinical application of Cell Therapeutics represents a new challenge for clinical scientists and all pharmaceutical applicants with regard to the current regulatory requirements for manufacturing, quality control and clinical trial approval in many parts of the world. The guidelines and standards are currently harmonized within the EU / FDA area of responsibility.

